# Prison Staff and Prisoner Views on a Prison Smoking Ban: Evidence From the Tobacco in Prisons Study

**DOI:** 10.1093/ntr/nty092

**Published:** 2018-05-26

**Authors:** Ashley Brown, Helen Sweeting, Greig Logan, Evangelia Demou, Kate Hunt

**Affiliations:** 1 Institute for Social Marketing, Faculty of Health Sciences and Sport, University of Stirling, Stirling, Scotland; 2 MRC/CSO Social and Public Health Sciences Unit, University of Glasgow, Glasgow, Scotland; 3 Faculty of Health Sciences and Sport, University of Stirling, Stirling, Scotland

## Abstract

**Introduction:**

In jurisdictions permitting prisoner smoking, rates are high (c75%), with smoking embedded in prison culture, leading to secondhand smoke exposures among staff and prisoners and challenges for smoking cessation. Momentum is building to ban smoking in prisons, but research on staff and prisoner views is lacking. We address this gap, providing evidence on staff and prisoner views throughout all Scottish prisons.

**Methods:**

Data were collected prior to the announcement of a (November 2018) prison smoking ban throughout Scotland. Mixed methods were used: surveys of staff (online, *N* = 1271, ~27%) and prisoners (questionnaire, *N* = 2512, ~34%); 17 focus groups and two paired interviews with staff in 14 prisons.

**Results:**

Staff were more positive than prisoners about bans and increased smoking restrictions, although prisoner views were more favorable should e-cigarettes be permitted. Nonsmokers were more positive than smokers. Whilst 74% staff and 22% prisoners agreed bans were a good idea, both groups acknowledged implementation and enforcement challenges. Staff views were influenced by beliefs about: acceptability of the policy in principle and whether/how bans could be achieved. Although some voiced doubts about smoke-free policies, staff likened a ban to other operational challenges. Staff raised concerns around needs for appropriate measures, resources and support, adequate lead-in time, and effective communication prior to a ban.

**Conclusion:**

We recommend that regular and open opportunities for dialogue within and between different stakeholder groups are created when preparing for prison smoking bans and that specific measures to address staff and prisoner concerns are incorporated into plans to create and maintain smoke-free environments.

**Implications:**

To our knowledge, this study is the first to research staff and prisoner views across a whole prison system prior to implementation of smoke-free policies. The results highlight potential challenges and suggest measures, which might help to maximize the success of bans. Our results are relevant for prison service managers responsible for the forthcoming introduction of a ban in Scottish prisons (November 2018) and for other prison systems and comparable institutions planning smoke-free initiatives. Given that prison smoking bans may be contentious, we recommend creating regular and open opportunities for dialogue between stakeholders when preparing for and maintaining smoke-free environments.

## Introduction

Smoking bans decrease exposure to secondhand smoke (SHS).^1^ In the United Kingdom, prisons had partial exemption from smoke-free legislation introduced in 2006/7. In the absence of smoke-free policies, prisoner smoking rates remained high and have been described as “one of the most pernicious public health problems affecting prisons…all too often…ignored [in] community based tobacco control policies.”^2^ The 2015 national biannual survey of prisoners in Scotland reported that 72% smoke,^3^ three times the national average and in line with figures for Europe (“64 to 88%” according to a European Commission report^4^), with little evidence of the reductions in smoking seen in the general population. This high prevalence partially reflects rates in deprived and socially excluded communities^5^ from which prisoners are disproportionately drawn. However, imprisonment can lead to uptake of, or increased, smoking,^6–11^ and high rates of smoking are reflected in high SHS levels within some prisons.^12,13^

WHO considers “there is no safe level of exposure to second-hand tobacco smoke,” citing evidence that SHS increases the risk of CHD, lung cancer, breast cancer, and respiratory symptoms and illnesses in adults, and, for those exposed during pregnancy, of low birth weight and preterm delivery.^14^ Both prisoners and staff (particularly those entering/opening prisoners’ cells) are potentially at risk and SHS exposure in prisons has attracted concern internationally. Momentum is building throughout the United Kingdom, as elsewhere (eg, Australia,^8^ USA^15^), to increase tobacco control or completely ban smoking in prisons, to improve staff and prisoner health and address health inequalities,^16,17^ although it has been suggested that smoking bans have been motivated “less by public health concerns than by fear of lawsuits from institutional staff and other inmates.”^18^ Several jurisdictions have implemented total smoke-free policies (ie, all indoor and outdoor areas) across their prison estate. New Zealand was the first to introduce total smoke-free prison policies country-wide in 2011,^19,20^ and measurements of indoor air quality in one prison before and after implementation showed “rapid and substantial” improvements.^21^ A systematic review which included three studies evaluating the cessation outcomes of an indoor smoking ban and seven (all United States) of complete smoking bans concluded that “a complete smoking ban (rather than partial ban) can effectively interrupt smoking behaviour”^22^ and an analysis of US data found prison tobacco control policies are associated with reduced mortaility.^2^ In the United Kingdom, total smoke-free policies were adopted by Broadmoor Secure Hospital in 2007, the Scottish State Hospital in 2011, Welsh prisons from January 2016, and at early adopter (and subsequently many more) prisons in England from March 2016. In July 2017, informed by evidence on SHS in Scottish prisons,^13^ the Scottish Prison Service (SPS) announced that Scotland’s prisons will be smoke-free from November 2018.

It is widely recognized, however, that making prisons smoke-free presents particular and considerable challenges. Butler described tobacco smoking as “an integral part of prison life and an established part of the prison culture,” serving a range of functions “as a surrogate currency, a means of social control, as a symbol of freedom in a group with few rights and privileges, a stress reliever and as a social lubricant.”^17^ Cigarettes can thus represent a means of dealing with the challenge of “killing time” and tobacco-based products offer prisoners “cultural capital to buy and exchange items; favours and protection,”^23^ as an alternative^24^ currency. The decision to smoke (or not) has been described as “one of the last functions that the inmate has control over”^23^ and its removal raises concerns about prisoner unrest.^25,26^ These concerns, rather than public health gains, have dominated much media coverage around the introduction of smoke-free prisons.^27^

To maximize the success and enforceability of smoke-free prison policy, it is crucial to understand how tobacco and smoking restrictions are viewed prior to, and in anticipation of, any policy change. To date, qualitative research on the meaning of smoking in prisons, and particularly on how this changes in the context of increased restrictions, is sparse. Two smaller^28,29^ and one larger qualitative studies,^30^ all conducted in the United States following the implementation of a partial/complete prison smoking ban, have noted the importance of policy “buy-in,” staff support and access to NRT. These studies reported the possibility of positive prisoner attitudes to a ban, while also highlighting the way in which a largely benign tobacco “market” can become problematic post-ban. However, no studies have undertaken a comprehensive overview of staff and prisoner views across a prison system.

We have addressed these gaps in research by presenting data from Phase 1 of the Tobacco in Prisons study (TIPs), a three-phase evaluation of the transition towards and implementation of smoke-free prisons in Scotland,^31^ Phase 1 data on objectively measured SHS from all 15 prisons are presented elsewhere.^13^ Here, we document the views of both prisoners and staff, drawing on survey and focus group/paired interview data collected several months prior to the announcement that Scotland prisons would be smoke-free from November 2018.

## Methods

TIPs Phase 1 data collection was designed to establish baseline values for smoking and cultural/social norms, in addition to levels of SHS,^13^ health indicators, and provision and experience of smoking cessation services, across all of Scotland’s 15 prisons. Phase 2 is ongoing and entails a process evaluation of initiatives, events, and changes in the period leading to implementation; Phase 3 will evaluate the impact of smoke-free policies.

Staff perspectives on smoking in prisons, smoking regulations, and smoking bans were collected via focus group discussions/paired interviews and an online questionnaire. Prisoner views were obtained via paper questionnaires. At the time of the data collection, prisoners were allowed to smoke in designated cells and outdoor spaces; staff and visitors were prohibited from smoking anywhere on prison grounds.

The protocol and study tools were approved by the Scottish Prison Service (SPS) Research Access and Ethics Committee and University of Glasgow’s College of Social Sciences Ethics Committee in August 2016 (ref number: 400150214). Research is independent of the SPS and Scottish Government; results are being fed back on an ongoing basis to all key stakeholders (eg, survey results feedback to the SPS TIPs Research Advisory Group, prisoners, staff, prison governors) to inform progress towards implementation.

### Staff and Prisoner Surveys

An invitation and link to the online staff questionnaire (live 1st November to 16th December 2016), plus reminders, were sent to an appointed contact within each prison who agreed to make this available to all prison officer, managerial and support staff within their prison. The questionnaire included sections on staff smoking, health, perceived SHS exposure, and opinions on smoking in prisons and prison smoking bans. The opinions items (detailed in Tables 1 and 2) were adapted from: a US survey of prison staff on restrictions to smoking in prisons;^32^ an Australian study of staff experience and attitudes to implementation of a smoke-free policy in a high security mental health in-patient facility;^33^ a Swiss survey of staff and patient attitudes to implementation of a smoke-free policy in a psychiatric hospital;^34^ and a Scottish study of bar workers’ attitudes to smoke-free public places legislation.^1,35^

**Table 1.** Prison Staff Opinions (% Agreeing/Strongly Agreeing) With Statements About Smoking in Prisons and Prison Smoking Bans, Overall and by Smoking Status

	Overall		According to smoking status - %			
	N/N	(%)	Current	Ex	Never	(sig)
**How much do you agree with these statements about smoking in prisons?** (five answer options collapsed to binary categories for analysis, “strongly agree” and “agree” vs. “no opinion,” “disagree,” and “strongly disagree”)						
Prison staff should be protected from cigarette smoke at work	1218/1270	95.9	82.1	96.6	98.2	(<0.001)
Prisoners who do not smoke should be protected from cigarette smoke	1206/1265	95.3	81.3	95.5	98.2	(<0.001)
There should be more NHS support for prisoners who want to stop smoking	879/1268	69.3	55.3	67.1	74.6	(<0.001)
Prisoners who smoke should not be forced to stop smoking	490/1268	38.6	68.0	42.0	28.8	(<0.001)
Prisoners who smoke are unlikely to ever stop long-term	537/1266	42.4	46.3	38.4	45.7	(0.028)
Smoking should not be allowed in any indoor areas of prisons	989/1259	78.6	42.1	75.6	89.5	(<0.001)
Smoking should not be allowed in any outdoor areas of prisons	480/1269	37.8	14.6	32.5	48.4	(<0.001)
**You have probably heard that smoking is no longer allowed in any areas (inside and outside) in prisons in some countries around the world like Canada, New Zealand, and Wales. What do you think of prison smoking bans like these?** (five answer options collapsed to binary categories for analysis, “strongly agree” and “agree” vs. “no opinion,” “disagree,” and “strongly disagree”)						
Prison smoking bans are a good idea	937/1268	73.9	35.0	69.1	87.4	(<0.001)
Prison smoking bans cause a lot of trouble (eg, prisoner fights, rioting, tobacco smuggling)	737/1269	58.1	75.4	60.7	51.7	(<0.001)
Prison smoking bans help prisoners stop smoking long-term (and after release)	640/1269	50.4	29.3	48.0	57.5	(<0.001)
Prison smoking bans are hard to enforce	780/1266	61.6	72.1	66.8	53.9	(<0.001)
Most prison staff want smoking bans	788/1269	62.1	36.6	55.4	74.6	(<0.001)
Prison smoking bans are OK if enough stop smoking support is available to prisoners	849/1266	67.1	40.2	64.7	75.3	(<0.001)
Prison smoking bans are OK if prisoners are allowed e-cigarettes or vapes	451/1270	35.5	36.6	37.0	33.7	(0.476)
**Would you be in favor of increased smoking restrictions in Scottish prisons?** (three answer options collapsed to binary categories for analysis, “in favour” vs. “no opinion” and “against”)	1004/1271	79.0	35.0	77.5	90.1	(<0.001)

**Table 2.** Prisoner Opinions (% Agreeing/Strongly Agreeing) With Statements About Smoking in Prisons and Prison Smoking Bans, Overall and by Smoking Status

	Overall		According to smoking status—%			
	N/N	(%)	Current	Ex	Never	(sig)
**How much do you agree with these statements about smoking in prisons?** (five answer options collapsed to binary categories for analysis, ‘strongly agree’ and ‘agree’ vs. ‘no opinion’, ‘disagree’, and ‘strongly disagree’)						
Prison staff should be protected from cigarette smoke at work	1337/2414	55.4	46.1	78.9	84.9	(<0.001)
Prisoners who don’t smoke should be protected from cigarette smoke	1644/2411	68.2	60.1	88.3	94.2	(<0.001)
There should be more NHS support for prisoners who want to stop smoking	1836/2404	76.4	76.5	74.7	77.6	(0.684)
Prisoners who smoke should not be forced to stop smoking	1900/2421	78.5	87.2	59.2	46.9	(<0.001)
Prisoners who smoke are unlikely to ever stop long-term	1042/2416	43.1	45.3	31.7	41.9	(<0.001)
Smoking should not be allowed in any indoor areas of prisons	1066/2408	44.3	33.1	68.6	84.5	(<0.001)
Smoking should not be allowed in any outdoor areas of prisons	432/2411	17.9	12.7	24.6	41.2	(<0.001)
**You have probably heard that smoking is no longer allowed in any areas (inside and outside) in prisons in some countries around the world like Canada, New Zealand, and Wales. What do you think of prison smoking bans like these?** (five answer options collapsed to binary categories for analysis, “strongly agree” and “agree” vs. “no opinion,” “disagree,” and “strongly disagree”)						
Prison smoking bans are a good idea	542/2415	22.4	10.2	48.8	67.1	(<0.001)
Prison smoking bans cause a lot of trouble (eg, prisoner fights, rioting, tobacco smuggling)	1954/2407	81.2	87.8	65.8	58.6	(<0.001)
Prison smoking bans help prisoners stop smoking long-term (and after release)	495/2394	20.7	14.3	34.8	43.6	(<0.001)
Prison smoking bans are hard to enforce	1545/2397	64.5	65.5	58.4	64.6	(0.047)
Most prison staff want smoking bans	782/2390	32.7	29.6	40.4	42.7	(<0.001)
Prison smoking bans are OK if enough stop smoking support is available to prisoners	877/2393	36.6	27.6	59.9	64.9	(<0.001)
Prison smoking bans are OK if prisoners are allowed e-cigarettes or vapes	1158/2395	48.4	47.7	49.7	50.3	(0.620)
**Would you be in favor of increased smoking restrictions in Scottish prisons?** (three answer options collapsed to binary categories for analysis, “in favour” vs. “no opinion” and “against”.	564/2395	23.5	11.3	50.0	67.1	(<0.001)

Results presented here are based on responses from 1271 prison-based staff (estimated 27% return) and include descriptive and bivariate analyses. The proportion of male respondents (71%) was identical to that of SPS staff overall.^36^ The proportion of smokers was somewhat lower (10% current, 23% ex, and 67% never/never regular smokers) than Scottish adults (21%, 25%, and 54%, respectively).^37^

Paper-based questionnaires (covering similar topics to those asked of staff) were distributed by TIPs research staff in three prisons; in the remaining 12 prisons questionnaires were handed to prisoners by prison staff at evening lock up and collected in sealed envelopes (to protect confidentiality) the next morning.

Descriptive and bivariate analyses of 2512 completed prisoner questionnaires (estimate 34% response) are presented. The proportion of smokers (74%) amongst prisoner responders was almost identical to that of prisoners overall (72%).^3^

### Focus Groups (and Paired Interviews)

From 14 prisons, 132 Scottish prison staff participated in a total of 19 qualitative data collection encounters (November 2016–April 2017). This included 17 focus groups and, for operational reasons, two paired interviews; on these two occasions, other staff were unable to attend at short notice and we proceeded to allow the people who came the opportunity to express their views. We indicated to “gatekeepers,” who facilitated recruitment within each prison, that we wished to include smokers and nonsmokers: 78 never smokers (NS), 27 ex-smokers (Ex), and 14 current tobacco cigarette or e-cigarette users (S) participated in focus groups (smoking status for 13 participants not known (NK)).

Focus groups (range = 5–12 participants; mode = 6) and paired interviews were led by a TIPs researcher using a topic guide which included sections on smoking and exposures to SHS, particularly within prisons; smoking norms and perceived prevalence within the prison; the “culture’ of smoking within prisons; management of nicotine addiction (including e-cigarettes) in prisons and wider society; and restrictions on smoking and opinions on these. The topic guide was designed to achieve an appropriate level of consistency for qualitative data collection; question wording was not prescribed. Participants were reminded that the researchers were independent of the SPS and Scottish Government, encouraged to express themselves freely and honestly, and invited to raise any points or views, which they thought to be pertinent. Discussions were audio recorded and transcribed verbatim with participants’ written consent; transcripts were reviewed for accuracy against the audio files and anonymized prior to data analysis. TIPs researchers who had conducted fieldwork read transcripts and agreed to a descriptive coding scheme: general tobacco and bans; prison bans; smoking culture in prisoners; smoking culture in staff; SHS exposures; e-cigarettes; quitting, alternatives, and cessation; operational, organizational, and local issues; Wales, England, and elsewhere; Scottish Prison Service and Scottish Government; and TIPs research. All transcripts were organized according to this coding scheme. Outputs from the “prison bans” code were then managed using the framework approach, facilitated by Nvivo software (QSR International). This process involved producing data summaries for every piece of coded data. Data summaries were displayed in a matrix format to facilitate analysis within and between focus groups/paired interviews. Data were thematically analyzed, ensuring that attention was paid to the range and diversity of views. Analytical summaries were compiled and reviewed in detail by at least one additional member of the authorship team and findings were checked by each member against a sub-sample of the transcripts. Illustrative extracts indicate the prison, focus group and speakers’ smoking status, for example, KA04 = prison K, group A, participant 04; NS = nonsmoker, Ex = exsmoker, S = smoker, NK = smoking status not known. Codes were randomly allocated to prisons by the research team.

## Results

### Levels of Staff and Prisoner Support for a Prison Smoking Ban: Survey Data

The percentage of staff and prisoners agreeing or strongly agreeing with a series of statements about smoking in prisons and comprehensive indoor/outdoor prison smoking bans (hereafter referred to as “prison smoking ban”) are shown in Tables 1 and 2. Perhaps unsurprisingly, staff indicated higher support for protection (for staff and prisoners) from SHS, restrictions of smoking, and smoking bans than prisoners. Thus almost all staff (strongly) agreed both that staff (96%) and nonsmoking prisoners (95%) should be protected from cigarette smoke whereas equivalent figures for prisoners were 55% and 68%; most staff (79%) but only a quarter (24%) of prisoners favored increased smoking restrictions in Scottish prisons; and, similarly, 74% of staff but only 22% of prisoners (strongly) agreed that “prison smoking bans are a good idea.” However, support for increased restrictions varied by smoking status in both staff and prisoners (as did almost all statements related to smoking in prisons), and were notably more positive among never smoking staff and prisoners (90% and 67%, respectively), than among current smoking staff and prisoners (35% and 11%). Nonetheless, many staff (58%) and most prisoners (81%) (strongly) agreed that “prison smoking bans cause a lot of trouble” and around two-thirds (62% staff, 65% prisoners) that bans may be “hard to enforce.” Notably, almost half of prisoners, irrespective of smoking status, (strongly) agreed that prison smoking bans “are OK if prisoners are allowed e-cigarettes or vapes.”

### Reasons Staff Supported or had Doubts About a Prison Smoking Ban

Analysis of the staff focus group/paired interview data suggested that perceptions of prison smoking bans were influenced by: (1) beliefs about whether a ban was acceptable in principle and (2) views on whether a ban could be successfully achieved. These are discussed below.

#### Beliefs About Whether a Ban was Acceptable in Principle

Views on whether a smoking ban was a fair and justifiable policy varied. Prisons were discussed as “homes” as well as workplaces, and concerns raised about restricting prisoner smoking while tobacco remains a legal substance in wider society.

(KA03[NS]) “Me personally, I’m a non-smoker, I’m an anti-smoker, I think it’s disgusting…but to say to somebody, you’re not getting to smoke in your own home, and it is their home, it’s their cell, sort of thing, I know it’s complicated with staff have got to access that as well, but I think it’s going to be a very, very difficult thing to justify… especially when it’s still a legal substance.”

Some staff thus regarded smoking as an unpleasant but expected hazard of working in a prison environment, as illustrated below:

HA02[S] …I understand the workplace ban….but this isn’t a normal job…I mean some of the stuff that comes your way working in this job you wouldn’t choose and you don’t welcome and all the rest of it, but it comes. It’s a risk and we manage that risk.

HA05[NS] …Working in [a prison] is totally different… people say it’s your place of work, aye it’s my place of work but it’s nothing like any other job…

Nonetheless, some voiced a need for alternative measures to limit SHS exposure, such as improved ventilation, and greater efforts to help prisoners to quit smoking.

By contrast, other staff expressed very strong views that SHS exposure at work was unacceptable, given its detrimental effect on health. Staff often described how tobacco smoke within prisons was unpleasant, even offensive, to experience. Some commented on the “*disgusting*” smell of smoke in prisons and complained how it could linger on hair and clothes after leaving work:

EA05[NS] “I go home from work and my husband will say oh you stink. And you don’t realise until you come out of the environment and when you don’t smoke and no one in your house smokes it’s horrible, your clothes are absolutely reeking, it’s really bad.”

Some groups voiced a sense of injustice that prison staff were not afforded the same legal protection from SHS as other workers, frustration with the decision to partially exempt prisons from smoke-free legislation introduced in Scotland in 2006, and a perception that there was insufficient regard to staff welfare:

BA03[NS] …the government says there’s no safe secondary smoke anywhere, but yet they’re quite content for every Scottish prison officer to go in that environment every day they’re at their work… it’s a routine part of their job but it’s an expected part…and that’s wrong….

BA01[Ex] Well the thing that’s wrong is the fact that we work in the only workplace where we’re expecting them to smoke and nobody else is expected to do that and that’s what’s wrong.

BA07[NS] There’s nobody. Can’t think of anybody

Furthermore, the complete prohibition on staff, but not prisoner, smoking on SPS premises was described by some as unfair.

#### Views on the Degree to Which a Ban Could be Successfully Achieved

All groups/paired interviews discussed whether and how a smoking ban could be successfully achieved in a prison environment if policies were to change, and views were again mixed. Current prison smoking culture, including perceptions that prisoners smoke in part to alleviate stress, anxiety and boredom, and possible defiance of a ban by prisoners unwilling or less able to quit smoking, were recognized as potential barriers to implementation. Implementation was expected to be particularly difficult for certain groups, such as new admissions and prisoners on remand or with mental health problems or drug addictions:

LA05[NS] “I struggle to see how we can be completely smoke free, because we’ve got prisoners in here that have been here for 20 years [who have] absolutely no interest in stopping smoking, guys that’s coming in on remand from the community, might only be here a few weeks, they’ve been smokers, they’re not going to quit within a few weeks…”

In contrast, reasons for believing a ban “could” be successfully implemented included the widespread public acceptance of tobacco restrictions in enclosed spaces, despite initial doubts about the policy, and introduction of bans in other challenging environments such as mental health hospitals. Thus, many staff believed prisoners would adapt to the ban, as they did to other prison rules, as illustrated in the following exchange in which a member of staff draws a parallel between the management of prisoners addicted to illegal drugs and those addicted to tobacco:

CA07[Ex] Because you could stop it in the jails, and everybody that’s been in the jail a while will get used to that. But if you’ve got somebody coming in for the first time, off the street, who’s a heavy smoker, how do we deal with that?

CA03[NS] They’re in a high stress situation, first time in jail, they’re maybe missing their families…they’ve got mental health issues, drugs…And then you’ve got to take their tobacco off them.

**Interviewer: So you think that’s almost like the last ‘domino’, yeah.
**

CA03[NS] Yeah, well, they’ll either hurt themselves, or try and hurt us, or hurt somebody else

CA06[NS] … I totally agree with what you’re saying... But then, at the same time, for those that are [addicted to] heroin, or cocaine… I know we have the detox, and methadone, but…they’re still not gonna get the same level, and they guys have to deal with it

CA07[Ex] That’s right.

CA06[NS] Because we can’t give them the illegal drugs that they’re used to… So it’s the same thing, it’s an addictive substance

All groups/paired interviews expressed concerns about potential negative consequences of a ban. These included: increased prisoner distress, self-harm, and suicide; episodes of unrest, violence, or riots; greater risks of physical or psychological harm to staff; creation of tobacco as contraband and associated problems such as smuggling, bullying, and debt; and use of alternatives (such as illicit tobacco, smoking tea bags, taking illegal substances) and associated problems such as using exposed wires from kettles as an ignition source. The extracts in Box 1 illustrate these concerns.

**Box 1: Staff views on potential unintended consequences of a ban**

Risks to prisoners and prison staff

“It would make a nicer working environment for us, but is that worth the backlash that would come as a result of that? It might make it a nicer place to work, but would it make it as safe a place to work? That’s what you’d have to weigh up, because it might affect your health in a different way.” (KA04[NS])

“Some prisoners who are just on the verge of taking their own lives, who can’t smoke...if they can’t smoke, how do we calm them down? If they’ve got nothing there to calm them down, it could tip them over the edge. So suicides will go up.” (NA06[NS])

Prisoner unrest

“There’s gonna be so many positives to it, with the ban, but there’s gonna be so many negatives, like you were saying, concerted [in]discipline in, obviously, your adult jails…” (CA06[NS])

“This blanket ban of smoking isn’t taking into account any of the ripple effect that it’s going to have, let alone the trouble it’s going to cause. Eventually we’ll manage the trouble in prisons like we’ve done before but it’s all the other things it’s not quite taking into account.” (CA02[S])

Contraband and associated problems

“… [if] we ban [tobacco] completely…It’ll still be smuggled in. It’ll become even more of a commodity than it is now…” (FA03[NS])

“I think you will also get an increase in the extent of bullying, because you have another commodity that’s become more valuable because you’ve made less of it, and…. I think you’ll increase in vulnerability because prisoners will be getting bullied for the tobacco, or even if they don’t smoke, they’ll be told, you will be buying tobacco for me this week at the canteen.” (LA04[NK])

Alternatives to tobacco, lighters and matches

“A blanket ban on smoking I can think of the knock-on effect of that. Everything from the health issues if guys are smoking cannabis which use tobacco, what will they do? They’ll move on to harder drugs, I’ll guarantee it.”(DA01[S])

“…when some of our prisons haven’t got tobacco in the halls here, they’ll smoke teabags. They’ve got to smoke something and the smell of tea bags, it’s actually worse than tobacco.” (HA03[N])

Nonetheless, some staff stated that such potential risks were *not* sufficient reason to reject smoking bans given the significant benefits to staff and prisoner health that could be achieved through making prisons smoke-free. Some challenged the view that major incidents, such as riots, might occur, as illustrated in the following extract where several participants agreed that a ban should be introduced despite any short-term difficulties it might create:

GC13[NS] …all you need to look at a…night in here right before they have got their canteen [shop from which prisoners can buy items]…and they have ran out of tobacco…it is a different shift in here… So, if you banned it…of course there would be issues, but I personally don’t think that that should be a reason to stop going ahead with it...

GC14[NS] To stop it, no.

GC13[NS] ...because [there’s] so many other risks to other people if you let them continue to smoke. So, I think obviously there would be problems, there would be potentially big problems, but I think eventually you would manage to get a grip of it and that’s a personal opinion.

GC14[NS] I’m with you…I think there’s always problems.

GC13[NS] It’s easy you can hide behind the fact that…if we stop this…this is going to be happening in jail, your job is going to be harder. I accept that, but I don’t think you can hide behind that…

#### Perceptions That a Ban was Inevitable

Staff perceived that, sooner or later, a ban was inevitable, not least because of Government aspirations to make Scotland smoke-free by 2034.^16^ In this context, staff spoke pragmatically about a ban, likening the task of implementation to other challenges faced at work. Thus, it was often stated in exchanges of simple “facts” that staff had the experience to “deal with” any problems associated with a ban, as they did with other challenges:

AA05[S] … it will be implemented and do you know what, it’ll be dealt with…

AA06[NS] We’ll deal with it ‘cause you’ve got to.

AA05[S] …and within a month it’ll be in and it’ll be no different. It’s like everything else. People stand up against it and they say this and they say that and do you know what, it just goes through and it gets done and before you know it, you’re going [saying], “Remember you used to smoke in jails!”

AA06[NS] ..it’s going to happen.

AA05[S] And it’ll happen and it’ll be done.

AA06[NS] And we’ll deal with it.

AA07[S] Exactly.

### Staff Views on Factors Important to Successful Implementation of a Future Ban

When asked what might contribute to successful implementation of a future smoking ban in Scottish prisons, staff identified several facilitators. These were: sufficient lead-in time; proactive and supportive management of the policy; adequate funding and other resources; effective consultation and communication with staff and prisoners; adequate prisoner smoking cessation support and measures to manage nicotine withdrawal; potentially piloting a ban prior to wider roll-out where appropriate; and learning from other jurisdictions which have gone smoke-free. These views are illustrated in Box 2.

**Box 2: Factors important to successful implementation of a future ban**

Sufficient lead-in time

“There would need to be a…reduction…not just…the ban’s coming in tomorrow. It’d need to be plenty of time, people being told, here’s the alternatives…essentially it would be a new sort of education for people….to say…come 18 months’ time you will not be allowed to smoke tobacco within anywhere in the jail. (IA11[S])

Proactive and supportive management of the policy

“…as long as you get the back up from the governors and management and says, right okay, we’ll deal with this. This might cause problems for the next six months, but we’ll deal with it because this is the way forward - we are not deviating from this. It is now a non-smoking jail. That’s it.” (GD04[N])

Adequate funding and other resources

“…if you did put a blanket ban on smoking inside the jail…if we were allowed to, we could be robust and strict, and we could…possibly prevent any major incidents of indiscipline. But we would need to be supported in that, we would need to be given the time, and we’d need to be given the resources to be able to deal with that.” (EA03[Ex])

“Where do then the cuts come from for everything else, like let’s try and manage the system that we have just now, find some money to do that before we like absolutely take this off the table, because the [health services] don’t have the money…” (KA05[S])

Effective consultation and communication with staff and prisoners

The communication part is key…In custody, great, but it also has to be outwith custody, in police cells, court houses… So that they’re aware it’s happening, that kind of seed is planted. (JA14[N])

“…staff quite often…things get handed down from on high…I think it’s really important that staff are involved at every stage, in what the alternatives might be”. (N04[NK])

“…get the staff buy-in and say, right, this is what we’re wanting to do, you guys are at the coalface, so to speak Will this work and if not, why won’t it work and what do we need to do to make it work? (MA02[NS])

“…I think…we have to try and make [prisoners] an important part of it, and say we’re not doing it because we are particularly fed up with opening doors and being stinking, but we’re actually more concerned that you are looking after your health.” (IA11[S])

Adequate prisoner smoking cessation support and withdrawal management

“I think, an admission process…is the key. Because you’re expecting somebody with an addiction, in the community, to then come into an area where, until such times as addiction recovery or support is put in place, they’re coming in and having to do cold turkey.” (CA01[NS])

“…a cigarette can really help them calm down and if they’re then told they’re not allowed to smoke and here are some patches, maybe an e-cig would be a good compromise because it would be a good similarity.” (MA01[Ex])

“They’re going to have to employ far more counsellors or people that are trained in trying to help people stop smoking…they’re going to have a lot more than four folk dealing with it.” (EA04[NS])

Potentially piloting a ban

“…Small steps. If you’re going to introduce something like this, it has to be small steps. Trial, did that work? No. What do we do next? It has to be introduced gradually.” (HA02[S])

Learning from other jurisdictions which have gone smoke-free

“If they can come up with what other people have done, take all the best bits from other people’s mistakes, and then say, right this is what we’re gonna do.” (NA01[NS])

However, there was debate between staff over the details of how a future ban should be implemented. For example, there was no obvious consensus on an optimum timescale from announcement to implementation. Suggestions generally ranged around 6–18 months, but some believed that 5 or more years’ preparation would be needed, whereas others, particularly those expressing a high degree of support for a smoking ban, called for a much shorter timescale (eg, days/weeks), likening exposure to SHS to other workplace hazards:

DA05[NS] …if this was asbestos we were talking about, would we say we’re going to wait another six months before we do anything about it? No, they would be shutting down this building… they’d be putting up special measures, control measures in place….

Others in this group, while recognizing the need to protect staff from SHS, favored a longer lead in time to ensure adequate cessation support for prisoners was in place:

Interviewer [addressing different group members who had expressed opinions on time scale] Are you saying “Monday”, and you’re saying “Maybe six months”?

DA05[NS]: I don’t think opinions come into it. It’s a matter of law.

DA03[NS]: I think you need to give folk time to be… prepared to actually support folk and be able to do it. It’s an addiction that they’ve got and that has to be considered…we’re still a caring profession although we should be caring for our staff and I totally agree with that…

DA05[NS]: Yes, we come first.

There were also diverse opinions on the need to phase in a ban prior to wider roll-out, either within designated residential areas of a single prison (eg, introducing voluntary smoke-free wings) or in designated prisons (eg, piloting of a smoking ban). In the following extract, staff discuss the pros and cons of introducing voluntary smoke-free wings after one participant suggested the idea:

GC14[NS]: Can staff volunteer to work in that environment?

GC11[S]: That would be it that would be the downside, somebody has got to work in the scabby smoking area.

[Conversation shifts topic, then returns to smoke-free wings]

GC16[NS]: I think it would be a good idea…if you maybe started off at the beginning by saying…‘This is a no smoking area’…see how the uptake was on [prisoners] wanting to go there and how it went…

GC14[NS]: But...how many people [prisoners] would manipulate that as well just to get a single cell …

GC13[NS]: But, I mean as soon as you caught anybody smoking in there [smoke-free wing] then they are shipped straight back. ..You would just say, “Right, well that’s you, you’re back then!”

Finally, there were discussions about the desirability or otherwise of introducing e-cigarettes into prisons as an alternative to tobacco. This issue is considered in more detail elsewhere; in brief, some staff regarded e-cigarettes as important to policy success, whereas others expressed concerns about their implications for staff and prisoner health and organizational safety.

There was some uncertainty about the extent to which adequate measures and support, as described above, would be in place prior to introduction of a ban in Scottish prisons. Specific fears were raised in respect of the need for: effective leadership of a ban (eg, sufficient consideration and management of risk); implementation strategies suited to local context (eg, adequate preparation time and avoidance of unnecessary delays in introducing a ban); and ways of working around constraints on public spending or the complexities of financing the support needed to help prisoners manage withdrawal and quit/abstain from smoking.

## Discussion

To our knowledge, this study is the first to research both staff and prisoner views across a country’s prison system prior to the announcement, or implementation, of a prison smoking ban. Using mixed methods, we found that opinions on a smoking ban differed between staff within and between prisons, between prisoners and staff, and also by smoking status. Focus groups and paired interviews with prison staff revealed that opinions were influenced by differing interpretations of: the legitimacy of restricting a prisoner’s freedom to smoke; the obstacles posed by current prisoner smoking culture; and the trade-off between health improvement and protection, potential physical and psychological risks to prisoners and staff, and threats to prison discipline. Consistent with previous studies,^19,38^ staff thought the success of prison smoking bans might depend on good governance and leadership; adequate time, support, and resources; good stakeholder communication and engagement; and effective management of nicotine addiction.

Strengths of this study include collection of data from staff *and* prisoners in *all* Scotland’s prisons, representing a range of prison environments and populations. However, the overall return rates to the staff and prisoner surveys were 27% and 34%, respectively. Thus, a degree of caution is required when generalizing from our results to the population of Scottish staff and prisoners. Although the sampling and recruitment approaches used for the questionnaires and focus groups were dictated by ethical, logistical, and operational considerations, and devised after extensive consultation with TIPs’ SPS-convened Research Advisory Group (which included representation from management, government, legal and health and safety staff, residential staff and union members), we recognize that participants were recruited using convenience sampling; those who volunteered to participate may not be representative of all Scottish staff and prisoners. In particular, it should be noted that a lower proportion of prison staff were smokers compared with the general population. However, Scottish prison staff have not been allowed to smoke anywhere on prison premises since 2008, so it is possible that rates of smoking in this group are actually lower than among Scottish adults, especially as movement through and out of a prison to smoke during a break may be considerably more difficult than in other workplaces. To our knowledge, no data exist to test this hypothesis. For operational reasons, paired interviews were conducted instead of focus groups on two occasions. While we acknowledge methodological differences between interviews and focus groups, we believe that these methods are complementary and can be combined effectively within a study. Logistical issues meant the present study could not explore prisoner attitudes qualitatively; this is planned for a subsequent phase of work. Finally, we acknowledge that levels of SHS vary greatly between and within prisons, no doubt influencing the strength of feelings and views in participants.

Novel contributions of this study are that it provides comprehensive and comparable evidence on how staff and prisoners view smoking bans prior to any decision on the introduction of smoke-free policy and highlights potential challenges to implementation as well as measures which might help to maximize success. Our results are timely and highly relevant for the forthcoming introduction of smoke-free prisons across Scotland in November 2018 and may be informative for other prison systems and comparable institutions planning smoke-free initiatives. In particular, the results highlight that the introduction of prison smoking bans removes an established activity and rare pleasure (sometimes even seen as a “right” or “privilege”) from individuals who are living in a difficult and often stressful environment. High rates of mental health problems in prisons may create further challenges in banning smoking, particularly as tobacco is (mistakenly) perceived to be effective in managing anxiety.^39^ Additionally, contextual factors such as increases in the prison population in recent decades,^40^ longstanding pressures on prison finance and staffing,^41^ and the relatively recent (2011) transfer of healthcare from Scottish prisons to health services^42^ have the potential to exacerbate problems in introducing smoke-free policy in Scotland’s prisons in November 2018. Although the scale of the task should not be underestimated, it is important to highlight that bans have been introduced into prison systems around the world experiencing common operating pressures, with evidence suggesting that implementation of smoke-free initiatives is often smoother than anticipated and fears of major unrest do not generally materialize.^19,38,43,44^

The findings of our study support the need for prison smoking bans to be accompanied by effective smoking cessation support, access to satisfactory tobacco alternatives and training for frontline staff on the effects of nicotine withdrawal and ideas for supporting quit attempts. In addition, there should be reviews of safeguarding procedures for vulnerable prisoners and increased promotion and investment in activities, which help to reduce anxiety, stress, and boredom. Ongoing measures will be needed for the maintenance of smoke-free environments, including continued strategies for management of nicotine addiction and fair and robust policing of bans.

Given that prison smoking bans may be contentious, we recommend that prison service managers create opportunities for regular and open dialogue within and between stakeholder groups. It is important that specific measures to address staff and prisoner concerns are incorporated into plans to bring about and maintain smoke-free environments.

## Funding

The TIPs study was funded by the National Institute for Health Research Public Health Research Programme (project number 15/55/44). The authors acknowledge funding from the Medical Research Council (MC_UU12017/12 and MC_PC_13027 to ED) and Chief Scientist Office (SPHSU12). The views and opinions expressed are those of the authors and do not necessarily reflect those of the Public Health Programme, NIHR, NHS, or the Department of Health.

## Declaration of Interests

None to declare.
